# Comparison of enzyme-linked immunosorbent assay and Fassisi^®^ bovine immunoglobulin G (IgG) immunoassay for quantification of bovine IgG in neonatal calf serum

**DOI:** 10.14202/vetworld.2021.3211-3215

**Published:** 2021-12-30

**Authors:** Marian Hampe, Stefanie Söllner-Donat, Klaus Failing, Axel Wehrend

**Affiliations:** 1 Clinic for Obstetrics, Gynecology, and Andrology of Large and Small Animals with Veterinary Ambulance, Justus-Liebig-University, D 35392 Giessen, Germany; 2 Animal Health Service, Thuringian Animal Diseases Fund, Jena, Germany; 3 Unit for Biomathematics and Data Processing, Justus-Liebig University, D 35392, Giessen, Germany

**Keywords:** antibody concentration, calves, colostrum, passive transfer, rapid method

## Abstract

**Background and Aim:**

Rapid tests are routinely used to estimate serum immunoglobulin G (IgG) concentrations in diagnosing a failure of passive transfer (FPT) in calves. The study aimed to compare the Fassisi^®^ Bovine IgG (FB-IgG) immunoassay and an enzyme-linked immunosorbent assay for quantifying bovine IgG in neonatal calf serum.

**Materials and Methods:**

A total of 277 calves of 1-10 days of age were used in this study. Blood samples were obtained, and serum was extracted by centrifuging the samples at 2740× *g* for 5 min at 20°C. The serum was analyzed using the FB-IgG according to the manufacturer’s specifications. Serum IgG concentrations were also determined by enzyme-linked immunosorbent assay (ELISA-IgG). FPT was defined as a serum IgG concentration <10 mg/mL.

**Results:**

The mean ELISA-IgG serum concentration was 8.40 mg/mL (SD=7.02, range=0.10-47.50 mg/mL). FPT prevalence based on the ELISA measurements was 66.8%. The prevalence of partial and full FPT based on the FB-IgG was 54.5%. The ELISA-IgG and FB-IgG results were subjected to correlation and regression analysis. Overall sensitivity and specificity of the FB-IgG were 61.1% and 58.7%, respectively. A statistically significant dependence on age was identified in the results.

**Conclusion:**

Our findings suggest that the FB-IgG rapid method is less accurate and provides no other advantages over established methods.

## Introduction

Timely and adequate uptake of colostral immunoglobulins (Ig) by calves after birth is critical for disease prevention and later milk production in dairy cows [1,2]. If the quality of the colostrum is poor, the calves may not receive sufficient levels of Ig – a condition referred to as failure of passive transfer (FPT) [3]. Therefore, early feeding programs were developed to provide sufficient high-quality colostrum to newborn calves [1,2,4,5]. Nevertheless, hypogammaglobulinemia due to FPT remains a significant problem [6].

Measurement of immunoglobulin G (IgG) concentrations in calf serum play a significant role in controlling and monitoring colostrum quality and supply. Direct measurement methods, however, are often complex, laboratory dependent, and expensive [7]. Thus, simpler and less costly indirect methods have been developed [8]. The precision, specificity, and sensitivity of these indirect methods, however, significantly differ from those of the reference method for direct measurement [8,9]. Therefore, subsequent efforts have focused on developing direct rapid tests and evaluating their suitability for routine use [10,11]. A rapid test based on a competitive immunoassay for validating FPT, the Fassisi^®^ Bovine IgG (FB-IgG) (Fassisi AT GmbH, Göttingen, Germany), has been available for several years. No independent scientific review has yet compared the effectiveness of the FB-IgG and enzyme-linked immunosorbent assay (ELISA)-IgG assays at determining IgG concentrations. The only two currently available verifications are provided in the manufacturer’s insert [12]. These studies, however, have not been independently validated.

The present study investigated the sensitivity and specificity of FPT detection using the FB-IgG compared to the reference method (ELISA-IgG). In addition, we examined the influence of calf health status and age on the FB-IgG results.

## Materials and Methods

### Ethical approval

This study was conducted in accordance with national laws and the methods approved by the Animal Welfare Committee of the competent regional authority (Regierungspraesidium) of Giessen, Germany, with the permission number kTV 08-2017.

### Study period and location

The study was conducted from February 2017 to March 2018. The study was conducted in the Middle-East of Germany (Hessen and Thuringia). The samples were processed at the Clinic for Obstetrics, Gynecology, and Andrology of Large and Small Animals with Veterinary Ambulance, Faculty of Veterinary Medicine, Justus-Liebig-University.

### Study population and sample collection

Blood samples were obtained from calves (n=277) ranging in age from 1 to 10 days, through the external jugular vein. Fiftysamples were obtained from the Clinic for Obstetrics, Gynecology, and Andrology of Large and Small Animals with Veterinary Ambulance, Justus-Liebig-University (Giessen, Germany) and 227 samples were obtained from 16 different farms in Hesse and Thuringia. Each animal was sampled only once. The calves were classified as healthy or sick by clinical examination, including measurement of rectal temperature (reference range=38.5-39.5°C), heartbeat (reference range=90-110 beats per min), and respiratory rate (reference range=30-45 breaths per min) [13]. In addition, calves were inspected for umbilical conditions and signs of dehydration. Calves displaying an undisturbed general condition and clinical parameters within reference ranges were classified as clinically healthy.

### Diagnostic testing

The IgG concentration was determined by laboratory sandwich ELISA [14,15] and the FB-IgG in all samples. Within 3 h of collection, the blood samples were centrifuged at 2740× *g* for 5 min at 20°C. The recovered serum was pipetted into sterile universal tubes. The IgG content was measured using the FB-IgG, after which the samples were frozen at –18°C. The frozen serum samples were sent to the Institute for Animal Protection, Behavioral Science, Animal Hygiene and Animal Husbandry in Munich, Germany, to determine the IgG concentration using sandwich ELISA [14,15]. FPT was defined as a serum IgG concentration below 10 mg/mL. The FB-IgG was performed strictly according to the manufacturer’s instructions, using the test cassettes, pipettes, and reagent vials provided in the respective kits. For the procedure, one drop (30 mL) of the serum was applied to the sample well of the cassette at room temperature (18-25°C) using the pipette and subsequently mixed with three drops of the reagent. The sample was observed to move spontaneously along the test track of the cassette by capillary action. After 10 min, the result was read. In addition to a permanently visible control line, a test line became clearly visible if the IgG concentration was determined to be <10 mg/mL. Under these conditions, the calf’s colostral IgG antibodies were considered to be severely depleted, indicating FPT, and the calf was assigned a score of 3. An invisible test line indicated that the sample contained an IgG concentration of ≥10 mg/mL, implying that successful and sufficient passive immunization was achieved, and the calf was assigned a score of 1. A faint test line indicated a borderline value, which was considered to be indicative of partial FPT [pFPT], and the respective calf was assigned a score of 2 [12].

### Statistical analysis

Statistical evaluation of the data was performed using theBMDP/Dynamic software package (Release 8.1, BMDP Statistical Software; University of California, LA, USA) [16]. The correlation between the IgG concentrations determined by ELISA-IgG and FB-IgG was determined using Spearman’s rank correlation coefficient (BMDP3D). The dependence of the results on age and health status was evaluated by logistic regression analysis, employing simple and multiple (including more than 1 independent variable) logistic regression models (BMDPLR). Determination of the sensitivity and specificity of the diagnosis of FPT was performed using a 2×2 table to assess the frequency of the true positive (sensitivity) and true negative (specificity) results of the assays (BMDP4F). The influence of age and health status on the results of the FB-IgG was also determined (BMDP4F). In all analyses, the ELISA-IgG values were logarithmically transformed to normalize the data distribution, which was systematically skewed to the right. The statistical significance level was set at a=0.05 unless otherwise indicated.

## Results

**Figure-1:**
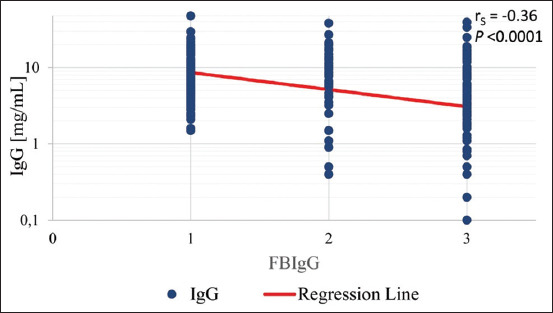
Correlation between the FB-immunoglobulin G (IgG) and ELISA-IgG concentration determinations. The equation for the regression line is: ELISA-IgG=10^(1.16 - 0.224 FB-IgG)^. IgG concentrations of calves that were sufficiently supplied with colostral antibodies are shown on the left (score 1, serum IgG concentration >10 mg/mL). Partial FPT with serum IgG concentration of 5–10 mg/mL was defined as a score of 2, and a complete FPT with serum IgG concentrations <5 mg/mL was scored with a value of 3, on the right of the graph.

The mean IgG concentration determined by ELISA-IgG was 8.40 mg/mL (range=0.10-47.50 mg/mL), and the distribution of the original values was skewed to the right. Among the 277 samples tested, the ELISA-IgG results indicated that 92 samples had an IgG concentration ≥10 mg/mL and 185 samples had an IgG concentration <10 mg/mL (indicating FPT). Thus, 66.8% of the calves examined had FPT according to the ELISA-IgG analysis. In comparison, the FB-IgG results indicated that 126 (45.5%) samples had a score of 1, 49 (17.7%) samples had a score of 2, and 102 (36.8%) samples had a score of 3. The FB-IgG results significantly correlated with the ELISA-IgG results (p<0.0001). However, Spearman’s rank correlation coefficient was low at r_s_=–0.36 (Figure-1). The equation of the regression line was: ELISA-IgG=10^(1.16-0.224 FB-IgG)^. In addition, a logistic regression model was used to examine the relationship between the simplified results of the FB-IgG (in which score values of 2 and 3 were considered to be a score of 0, indicating inadequate supply of IgG, and a score of 1 indicated adequate supply) and the logarithm of the ELISA-IgG level of the serum samples. This analysis revealed a highly significant (p<0.0001) relationship between the two assay procedures with a model coefficient of 1.839 and a resulting odds ratio (OR) of 6.29 (95% confidence interval=3.22≤OR≤12.3). The logistic regression equation was: Y=exp[–1.60+1.839˙lgELISA]/(1+ exp[–1.60+1.839˙lgELISA]). This equation models the probability of detecting an adequately supplied calf independent of the true ELISA-IgG concentration. The OR value revealed a 6.29-fold increase in the odds that a calf will be determined to be adequately supplied based on the FB-IgG if the ELISA-IgG concentration is greater by 1 decimal.

To determine the sensitivity and specificity of the FB-IgG, the pFPT (score value=2) and FPT (score value=3) results (n=116) were once more combined into a single group and compared with the sufficiently supplied (score=1) values. The FB-IgG correctly identified inadequately supplied calves with an estimated sensitivity of 61.1% and an estimated specificity of 58.7% (Table-1). We performed a multiple logistic regression analysis to assess the possible additional influences of age and health status simultaneously between the ELISA-IgG and the FB-IgG. Of the 277 calves sampled, 202 were classified as healthy and 75 were classified as sick (including bronchopneumonia, umbilical inflammation, and diarrhea). Age significantly affected the results of the FB-IgG (p<0.0001), whereas health status did not (p=0.98). As a result, the FB-IgG indicated that the calves were sufficiently supplied (score=1) more frequently in older animals, independent of the ELISA-IgG results. For each additional day of life, the probability of being classified as having received a sufficient IgG supply increased by an estimated OR factor of 1.22 (95% confidence interval=1.11≤OR≤1.34). To confirm this result, the calves were categorized into four subgroups on the basis of age and health status: Age ≤3 d or age >3 d, and healthy or ill (Table-2). Within this age range, complete gut closure can be assumed. The proportion of healthy calves correctly classified as being inadequately supplied (sensitivity) decreased from 79.7% (age ≤3 d) to 38.6% (age >3 d), while that in sick animals decreased from 83.3% (age ≤3 d) to 53.8% (age >3 d). The specificity in healthy calves increased from 48.4% (age ≤3 d) to 61.9% (age >3 d), while that in sick calves increased from 66.7% (age ≤3 d) to 71.4% (age >3 d).

**Table 1:** Results of the FB-IgG and ELISA-IgG assays in calves (up to 10 days of age).

	ELISA-IgG (gold standard/reference test) (n/%)		
FB-IgG (n/%)	113/61.1 True positive (sensitivity)	38/41.3 False positive	151 (FB-IgG test positive)
	72/38.9 False negative	54/58.7 True negative (specificity)	126 (FB-IgG test negative)
	185/100 (FPT/pFPT positive)	92/100 (non-FPT)	277

*FPT positive=<10 mg/mL; FPT negative=≥10 mg/mL; n=Absolute number; %=Percentage value. IgG=Immunoglobulin G, FPT=Failure of passive transfer, ELISA=Enzyme-linked immunosorbent assay

**Table 2:** Comparison of the classification between the FB-IgG and ELISA-IgG of the calves categorized by age and health status.

		ELISA-IgG (gold standard/reference test) (n/%)		
Age≤3 days, healthy	FB-IgG (n/%)	47/79.7 True positive	16/51.6 False positive	63 (FB-IgG test positive)
		12/20.3 False negative	15/48.4 True negative	27 (FB-IgG test negative)
		59/100 (FPT/pFPT positive)	31/100 (non-FPT)	90
Age≤3 days, sick	FB-IgG (n/%)	25/83.3 True positive	4/33.3 False positive	29 (FB-IgG test positive)
		5/16.7 False negative	8/66.7 True negative	13 (FB-IgG test negative)
		30/100 (FPT/pFPT positive)	12/100 (non-FPT)	42
Age 4-10 days, healthy	FB-IgG (n/%)	27/38.6 True positive	16/38.1 False positive	43 (FB-IgG test positive)
		43/61.4 False negative	26/61.9 True negative	69 (FB-IgG test negative)
		70/100 (FPT/pFPT positive)	42/100 (non-FPT)	112
Age 4-10 days, sick	FB-IgG (n/%)	14/53.8 True positive	2/28.6 False positive	16 (FB-IgG test positive)
		12/46.2 False negative	5/71.4 True negative	17 (FB-IgG test negative)
		26/100 (FPT/pFPT positive)	7/100 (non-FPT)	33

*FPT positive=<10 mg/mL; FPT negative=≥10 mg/mL; n=Absolute number; %=Percentage value. pFPT=Partial failure of passive transfer, FPT=Failure of passive transfer, serum IgG concentration<10 mg/mL=Hypogammaglobulinemia, serum IgG concentration≥10 mg/mL=Sufficient supply of colostral antibodies, n=Absolute frequency, %=Relative frequency. IgG=Immunoglobulin G, ELISA=Enzyme-linked immunosorbent assay

## Discussion

The present study compared the accuracy of the FB-IgG and ELISA-IgG in assessing serum IgG levels to diagnose FPT in calves. Although the rapid method and the ELISA-IgG results were highly significantly correlated, the strength of the correlation was only moderate (r_s_=–0.36). In this study, FPT was defined as an IgG value of ≤10 mg/mL, which is consistent with the recommendation of the test manufacturer and published literature [5-11]. Thus, a negative assay result indicated that the calf had received an adequate supply of IgG. Although the choice of the threshold value was arbitrary, it has a clear precedent [5,9]. Regardless, it is important to note that IgG concentrations higher than 10 mg/mL may be required to ensure optimal calf health under poor sanitary conditions.

Conversely, lower IgG concentrations may be sufficient under excellent sanitary conditions. For diagnosing FPT with the FB-IgG, a sensitivity of 61.1% and a specificity of 58.7% were found when considering all calves. Consequently, the rapid test incorrectly classified 39.7% (110/277) of the calves assayed. When the age classes were evaluated separately, 72.0% of calves up to 3 days old and 49.7% between 4 and 10 days old were correctly classified. The data provided in the FB-IgG instructions appear to be inconsistent with the data obtained in the present study. Two studies are cited in the FB-IgG documentation: A 2014 study performed at the University of Goettingen and a 2014 study by “ID Ringbox IgG” [12]. These two studies reported sensitivities of 97.7% and 99.9% and specificities of 92.4% and 93.2%, respectively, for this assay. In the absence of the original published data (full references were not provided), we cannot speculate on the reasons for the significant difference between our results and those of the other two studies. As the FB-IgG directly measures the IgG concentration in the blood, the results would be expected to agree with those of the ELISA-IgG determination, irrespective of the calf’s age and disease state. Our findings indicated that the state of health had no significant influence on the results of either method. To determine the influence of age, the subjects were stratified into two groups according to their age at sampling. Unexpectedly, the experimental difference between age groups was statistically significant, indicating a correlation with age. With increasing age, the FB-IgG classified significantly more calves than the ELISA-IgG as sufficiently supplied with colostral IgG. In the group of calves up to 3 days old, numerous animals had not yet ingested colostrum at the time of sampling and, accordingly, had low serum IgG concentrations. The FB-IgG rapid test correctly identified many of these calves as having deficient immunoglobulin concentrations in the serum. The serum IgG concentrations in the older calves with FPT had increased significantly (but were still ≤10 mg/mL) and thus were close to the values of the adequately supplied young animals. Therefore, the risk of misclassification was increased due to the sample concentration being much closer to the threshold value. The strict requirement to read the FB-IgG test results at precisely the 10 min mark presents a significant limitation. If the assay time extends beyond 10 min, the test indicator lines may weaken, leading to misclassification of the calf. The results of the ELISA-IgG significantly correlated with those of the FB-IgG rapid method. Our findings, however, indicate that the FB-IgG rapid method does not fulfill the requirements for monitoring passive immunization in newborn calves.

## Conclusion

Therefore, we conclude that established indirect rapid methods, such as total serum protein determination or measurement of the serum gamma-glutamyl transferase activity, provide significantly more reliable results than the FB-IgG rapid method for detecting FPT in calves.

## Authors’ Contributions

MH: Performed animal experimentation, biochemical assays, analyzed and interpreted the results, and wrote the manuscript. SS: Organized the implementation of blood sampling on farms in Thuringia. KF: Performed the statistical analysis. AW: Designed the study, coordinated the research activity planning, and edited the manuscript. All authors read and approved the final manuscript.
